# High-Intensity Intermittent Swimming Improves Cardiovascular Health Status for Women with Mild Hypertension

**DOI:** 10.1155/2014/728289

**Published:** 2014-04-10

**Authors:** Magni Mohr, Nikolai Baastrup Nordsborg, Annika Lindenskov, Hildigunn Steinholm, Hans Petur Nielsen, Jann Mortensen, Pal Weihe, Peter Krustrup

**Affiliations:** ^1^Sport and Health Sciences, College of Life and Environmental Sciences, St. Luke's Campus, University of Exeter, Exeter EX12LU, UK; ^2^Department of Food and Nutrition, and Sport Sciences, University of Gothenburg, 405 30 Gothenburg, Sweden; ^3^Department of Nutrition, Exercise and Sports, Copenhagen Centre for Team Sport and Health, University of Copenhagen, 2100 Copenhagen, Denmark; ^4^The Faroese Confederation of Sports and Olympic Committee, 100 Torshavn, Faroe Islands; ^5^Department of Nursing, Faculty of Natural and Health Sciences, University of the Faroe Islands, 100 Torshavn, Faroe Islands; ^6^Southern Hospital, The Faroese Hospital System, Faroe Islands; ^7^Department of Medicine, The Faroese National Hospital, 100 Torshavn, Faroe Islands; ^8^Department of Clinical Physiology, Nuclear Medicine & PET, Rigshospitalet, Copenhagen University Hospital, 2100 Copenhagen, Denmark; ^9^Department of Occupational Medicine and Public Health, The Faroese Hospital System, 100 Torshavn, Faroe Islands

## Abstract

To test the hypothesis that high-intensity swim training improves cardiovascular health status in sedentary premenopausal women with mild hypertension, sixty-two women were randomized into high-intensity (*n* = 21; HIT), moderate-intensity (*n* = 21; MOD), and control groups (*n* = 20; CON). HIT performed 6–10 × 30 s all-out swimming interspersed by 2 min recovery and MOD swam continuously for 1 h at moderate intensity for a 15-week period completing in total 44 ± 1 and 43 ± 1 sessions, respectively. In CON, all measured variables were similar before and after the intervention period. Systolic BP decreased (*P* < 0.05) by 6 ± 1 and 4 ± 1 mmHg in HIT and MOD; respectively. Resting heart rate declined (*P* < 0.05) by 5 ± 1 bpm both in HIT and MOD, fat mass decreased (*P* < 0.05) by 1.1 ± 0.2 and 2.2 ± 0.3 kg, respectively, while the blood lipid profile was unaltered. In HIT and MOD, performance improved (*P* < 0.05) for a maximal 10 min swim (13 ± 3% and 22 ± 3%), interval swimming (23 ± 3% and 8 ± 3%), and Yo-Yo IE1 running performance (58 ± 5% and 45 ± 4%). In conclusion, high-intensity intermittent swimming is an effective training strategy to improve cardiovascular health and physical performance in sedentary women with mild hypertension. Adaptations are similar with high- and moderate-intensity training, despite markedly less total time spent and distance covered in the high-intensity group.

## 1. Introduction

Arterial hypertension is associated with cardiovascular morbidity and mortality, and it is well known that the risk of arterial hypertension is markedly elevated by obesity and an inactive lifestyle [1, 2]. Additionally, there is strong evidence that exercise training lowers arterial blood pressure, improves aerobic fitness, and counteracts several other cardiovascular risk factors related to increased morbidity in patients with mild to moderate hypertension [3, 4], but it is still debated whether the magnitude of training response is related to exercise mode and the type of training performed.

The vast majority of studies investigating the relationship between exercise training and cardiovascular health responses have applied running, cycling, or team sports participation as the training intervention [5–7], whereas few have examined the effects of different aquatic exercise regimes [8–10]. Swimming may be considered a good choice of training especially for obese middle-aged and elderly individuals because it involves minimum weight-bearing stress, which may reduce the risk of injury. In addition, swimming engages the upper body musculature where the potential for metabolic adaptation can be hypothesized to be larger than in the postural musculature. However, little information is available concerning the effects of regular swimming exercise training on the cardiovascular health profile. Nualnim and coworkers [10] demonstrated that 12 wks of regular 15–45 min continuous moderate-intensity swimming lowered systolic blood pressure (SBP) by 9 mmHg in adults older than 50 yrs with mild hypertension. The swimming exercise training also resulted in a 21% increase in carotid artery compliance, as well as improvement in flow-mediated dilation and cardiovagal baroreflex sensitivity [10]. However, no studies have compared different swim training regimes in sedentary women suffering from mild to moderate arterial hypertension.

Lack of time is a common explanation why people fail to participate continuously in traditional exercise regimes based on prolonged session of moderate-intensity training. Therefore, it is of interest to explore the health effects of short-duration exercise training protocols. Numerous findings indicate that brief high-intensity training appears to be efficient in improving aerobic fitness and other physiological adaptations of importance for the cardiovascular health status in untrained individuals [6, 7, 11]. Moreover, short-term sprint training apparently provoked similar muscle metabolic and exercise performance adaptations as prolonged submaximal training protocols [12, 13]. These studies challenge the pronouncement by sports medicine authorities that 150–250 min of moderate-intensity exercise per week is required to maintain a healthy lifestyle [14, 15] and support the idea that 75 min of vigorous exercise may be sufficient [16]. For example, Nybo et al. [6] found differences in the adaptive response within several indicators of cardiovascular health to short-duration high-intensity intermittent running compared to prolonged submaximal continuous running, including more pronounced effects on maximal oxygen uptake for the high-intensity training group. This study was performed on sedentary men, while Metcalfe et al. [17] demonstrated marked improvements in aerobic capacity and metabolic health after intensified cycling in sedentary participants of both genders. However, it is currently unclear to what extent women respond to submaximal prolonged versus short-term high-intensity swim training. Gender differences have been shown to be present within a range of physiological adaptations to exercise training [4, 18]. For example, women appear to display smaller reductions in blood pressure after exercise training interventions in comparison to their male counterparts [4, 5, 19]. It is therefore of importance to investigate the effect of two types of swimming exercise training on the cardiovascular disease risk profile in sedentary women with mild to moderate hypertension.

Thus, the objective of the present study was to test the hypothesis that high-intensity swim training is an efficient strategy to reduce blood pressure and improve the cardiovascular health profile in sedentary premenopausal women with mild to moderate hypertension.

## 2. Materials and Methods

Sixty-two sedentary premenopausal women with mild to moderate arterial hypertension were recruited for the study. The subjects were selected among 262 volunteers based on training history, medication, blood pressure, and body mass index. A total of 83 participants were recruited. 62 took part in the present study and 21 were randomly assigned to a football group being part of another study [20]. In addition, the control group (20 participants) in the present study was also the controls in the above-mentioned study by Mohr et al. [20]. The study was approved by the ethical committee of the Faroe Islands as well as the Sport and Health Sciences Research Ethics Committee at the University of Exeter, Exeter, UK, and conducted in accordance with the Declaration of Helsinki (1964). After being informed verbally and in writing of the experimental procedures and associated risks, all participants gave their written consent to take part in the study.

### 2.1. Experiment Design

The study was designed as a randomized controlled trial. After initial testing of the 262 volunteers, 62 participants were enrolled in the present study based on selection criteria being a sedentary lifestyle for the last two years, mild hypertension (mean arterial pressure 96–110 mmHg), and a body mass index >25. Participants treated with adrenergic beta-antagonists were excluded. Participants using diuretics and ACE inhibitors (*n* = 4) were not excluded from the study, but none of the four subjects changed their medication during the intervention period. The participants were randomized into a high-intensity intermittent swimming training group (HIT: age 44 ± 2 (36–49) ±SEM (range) yrs; height 164 ± 1 cm; weight 76.5 ± 1.9 kg; *n* = 21), a moderate-intensity continuous swimming group (MOD: age 46 ± 2 (38–48) yrs, height 165 ± 1 cm, weight 83.8 ± 4.3 kg; *n* = 21), and a control group (CON: age 45 ± 2 (35–48) yrs, height 166 ± 1 cm, weight 76.4 ± 2.6 kg; *n* = 20). The training groups took part in two types of swimming training with 3 training sessions per week for 15 wks, while CON had no training or lifestyle changes in the same period. There were no dropouts from the study, but one subject in the MOD group suffered from aquatic phobia and was therefore moved to CON. All subjects performed an intermittent swimming sprint test and an endurance swimming test, as well as an intermittent running test with heart rate recordings, and had their blood pressure, resting heart rate (RHR), body fat content, and blood cholesterol measured before and after the intervention. Finally, basic anthropometrical measurements were performed. The pre- and posttests were conducted in the same order. The postfitness tests were conducted 48–72 h after the last training session. The training was continued until the last measurement was obtained. The dietary intake was not controlled during the training period and the testing periods were not timed in relation to the menstrual cycle.

### 2.2. Training Intervention

The HIT participants completed in total 44 ± 1 (39–50) training sessions over the 15-week intervention period corresponding to 2.9 ± 0.1 (2.6–3.3) sessions per week. Every session lasted ~15–25 min (3–5 min of effective swimming) and consisted of 6–10 30 s all-out free-style swimming (front crawl) intervals interspersed by 2 min of passive recovery after training principles previously described [21, 22]. In the first 6 wks of training the participants completed 6 intervals, the following 6 wks included 8 intervals, and the final 3 wks consisted of 10 all-out swimming intervals. The MOD group completed a total of 43 ± 1 (37–49) training sessions over 15 wks corresponding to 2.9 ± 0.1 (2.5–3.3) training sessions per week. All MOD training sessions lasted 1 h and consisted of continuous front crawl swimming where the participants were encouraged to swim as far as possible in every session. Five trained swimming coaches were present during all training sessions in order to give technical advice and control the intensity and duration of the training and to secure a safe training environment. Heart rate was measured during one training session in week 1 and one session in week 15 of the training intervention, and the swimming distance was noted in every session.

### 2.3. Blood Pressure and RHR Measurements

The participants reported to the hospital at 8:00 a.m. after an overnight fast and rested in a supine position for 2 h. Systolic blood pressure (SBP) and diastolic blood pressure (DBP) were measured according to standard procedures [23] using an automatic BP monitor (HEM-709; OMRON, IL, USA) once every 30 min over the 2 h resting period. The average of the four measurements was used as the test result. Mean arterial pressure (MAP) was calculated as 1/3 SBP + 2/3 DBP. Resting HR was measured during the same time intervals as the BP recordings.

### 2.4. Resting Blood Sampling

A resting blood sample was collected under standardized conditions from an antecubital vein between 7:00 and 8:00 a.m. after an overnight fast using venipuncture technique. The blood was rapidly centrifuged for 30 s and analyzed by an automatic analyzer (Cobas Fara, Roche, France) using enzymatic kits (Roche Diagnostics, Germany) for determination of total cholesterol, LDL-cholesterol, HDL-cholesterol, and triglyceride levels.

### 2.5. DXA Scanning

Whole-body body fat and lean body mass were evaluated by total body DXA scanning (Norland XR-800, Norland Corporation, Norway). The body was segmented in accordance with standard procedures to evaluate regional fat distribution [24], and all analyses were performed using Illuminatus DXA software (Norland Corporation, Norway). The effective radiation dose was <0.2 mSv per scan.

### 2.6. Exercise Performance Testing

The participants in HIT and MOD performed two front crawl swimming tests before and after intervention. To evaluate if the high-intensity training improved the ability to repeatedly perform high-intensity swimming more than moderate-intensity training, a repeated swimming sprint test (RSST) composed of 4 × 25 m sprinting starting every 60 s was performed. The participants were instructed to swim each 25 m as fast as possible. To evaluate if continuous training was more efficient in improving continuous swimming performance, a 10 min continuous swimming test was performed. Swimmers were instructed to complete the largest possible swimming distance during the 10 min. Swim testing was performed in a 25 m pool at a water temperature of 26°C. To evaluate if the swim-training intervention improved exercise capacity in a land-based activity, all participants additionally completed a shuttle-run test. The Yo-Yo Intermittent Endurance test, level 1 (Yo-Yo IE1), was completed before and after the training period. The Yo-Yo IE1 test consists of 2 × 20 m shuttle runs interspersed by a 5 s recovery period consisting of 2 × 2.5 m jogging (see [25]). There is a gradual speed progression during the test, which is controlled by a CD player [26]. The participants run until the point of exhaustion defined as the second time they are unable to complete the 2 × 20 m runs at the required pace [25]. Maximum heart rate (HR_max⁡_) was determined during the test as previously described [25]. The preintervention test was performed within ten days of the first training and the postintervention test four days after the last training session. The tests were conducted indoor on a wooden surface at environmental temperatures between 18 and 20°C. The tests were preceded by a short warm-up period consisting of the first three of the 2 × 20 m shuttle runs, followed by a 2 min recovery period before the exhaustive test. Heart rate was measured continuously during the tests using Polar Vantage NV chest belt monitor weighing ~100 g (Polar Electro Oy, Kempele, Finland), and HR_max⁡_ was determined as previously described [26]. The pre-and postintervention tests were conducted at the same time of day. All participants were familiarized to all test procedures prior to the experiment according to guidelines presented in Bradley et al. [26]. The participants were instructed to avoid exercise training and intake of alcohol the day prior to the testing and nutritional items rich in caffeine on the day of testing. In addition, the participants were also instructed to note the food intake and follow similar nutritional guidelines during the last 24 h before both test periods.

### 2.7. Hip and Waist Circumference and Body Weight

Hip and waist circumference was assessed as described by Kharal et al. [27]. Body mass was assessed by weighing the participant. The weighing was performed in the morning after an overnight fast using a platform scale (Ohaus, Germany).

### 2.8. Statistical Analyses

Data are presented as means ± SEM. Between- and within-group data were evaluated both by two-factor mixed ANOVA design and with one-way ANOVA on repeated measurements. When a significant interaction was detected, data were subsequently analyzed using a Newman-Keuls post hoctest. Significance level was *P* < 0.05.

## 3. Results

### 3.1. Heart Rate and Distance Covered during Training

Average mean and peak HR during HIT training in the first and last weeks of the intervention was 158 ± 5 and 176 ± 2 bpm, respectively, corresponding to 85.5 ± 1.1 and 95.3 ± 1.1% HR_max⁡_, respectively, which was higher (*P* < 0.05) than average values in MOD (132 ± 4 and 144 ± 3 bpm equivalent to 72.5 ± 0.9 and 79.1 ± 1.0% HR_max⁡_). No differences in heart rate between the first and last weeks of training were detectable within either training group. In HIT the average swim distance per session during the first week was 131 ± 7 m and increased (*P* < 0.05) to 269 ± 10 m during the last training week. Average swim distance per swimming interval increased (*P* < 0.05) by 28 ± 6% from the first to the last training week. In MOD the average swim distance per session was 1177 ± 41 m during the first training week and was increased (*P* < 0.05) to 1787 ± 35 m (52.8 ± 3.2%) during the last training week.

### 3.2. Blood Pressure and Resting Heart Rate

**Figure 1:**
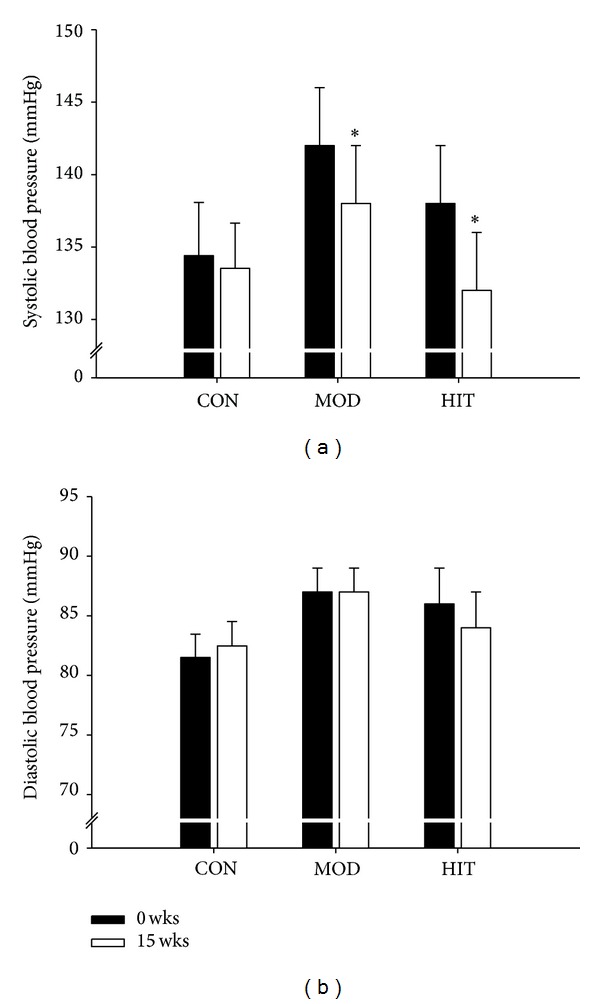
Systolic and diastolic blood pressure before and after 15 wks with thrice-weekly training sessions of high-intensity swimming (HIT) and moderate-intensity swimming (MOD) in comparison to an inactive control group (CON). Data are presented as means ± SEM. # denotes significant within-group differences. ∗ denotes significant difference between the training groups and CON.

Prior to the intervention period, SBP and DBP were 138 ± 4 and 86 ± 3 mmHg in HIT, 142 ± 4 and 87 ± 2 mmHg in MOD, and 134 ± 4 and 82 ± 2 mmHg in CON, respectively, with no differences between groups. In HIT, SBP decreased (*P* < 0.05) by 6 ± 1 mmHg (4 ± 1%) during the 15 wk intervention period (Figure 1), while the MOD group displayed a decrease (*P* < 0.05) of 4 ± 1 mmHg (3 ± 1%) in SBP. DBP was similar before and after intervention for HIT and MOD (Figure 1). No significant changes took place in neither SBP nor DBP in CON (0 ± 0 and 0 ± 0 mmHg, Figure 1). MAP was 103 ± 4 and 99 ± 2 mmHg before training in HIT and CON, respectively, and tended (*P* = 0.06) to decrease (3 ± 1 mmHg, 3 ± 1%) after intervention in HIT, with no changes in MOD or CON (−1 ± 0 and 0 ± 0 mmHg, Figure 1). Sixteen of the twenty-one subjects in HIT experienced a decline in MAP during the intervention period, with corresponding numbers in MOD being thirteen out of twenty-one and eleven out of twenty in CON.

Resting HR decreased (*P* < 0.05) by 5 ± 1 bpm over 15 wks both in HIT (76 ± 2 to 71 ± 2 bpm) and MOD (78 ± 3 to 73 ± 2 bpm), whereas it was not significantly altered in CON (77 ± 2 and 74 ± 2 bpm).

### 3.3. Body Fat, Lean Body Mass, and Anthropometry

**Figure 2:**
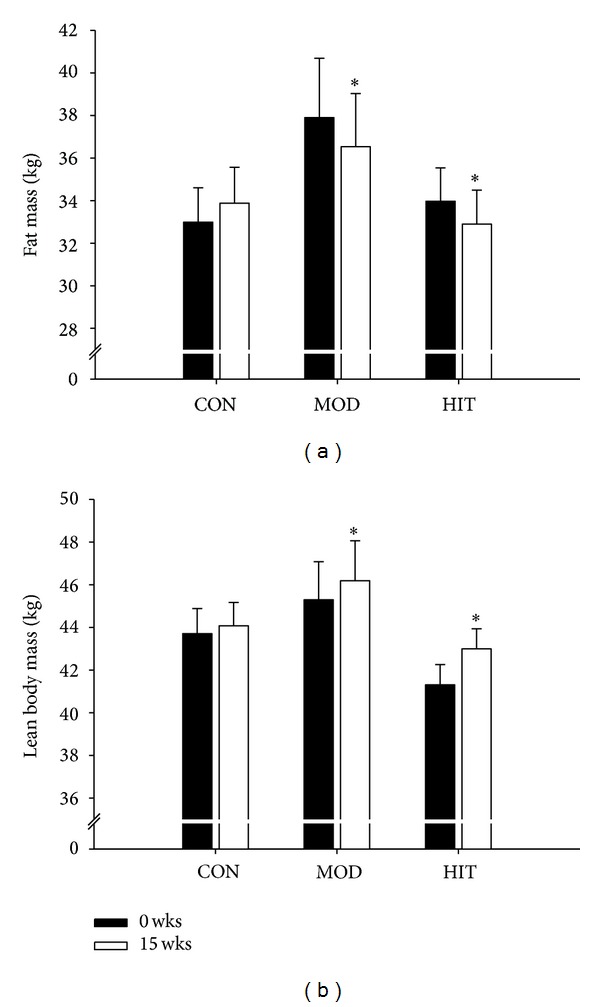
Changes in body composition, including fat mass, lean body mass, and total body weight after 15 wks with thrice-weekly training sessions of high-intensity swimming (HIT) and moderate-intensity swimming (MOD) in comparison to an inactive control group (CON). Data are presented as means ± SEM. ∗ denotes significant difference between the training groups and CON.

Total body fat percentage was 43.1 ± 1.1, 44.1 ± 1.2, and 41.0 ± 1.2% before training in HIT, MOD, and CON, respectively, and decreased (*P* < 0.05) by a similar magnitude to 41.4 ± 1.2 and 42.1 ± 1.0% in HIT and MOD, respectively, with no change in CON (41.5 ± 1.1%). Total fat mass decreased by 1.1 ± 0.2 and 2.2 ± 0.3 kg (*P* < 0.05) in HIT and MOD, respectively, during the 15 wks but remained similar in CON (Figure 2). Lean body mass increased (*P* < 0.05) by 1.7 ± 0.3 and 1.3 ± 0.3 kg in HIT and MOD, respectively, with no significant changes in CON (Figure 2). Hip circumference was lowered (*P* < 0.05) in MOD (108 ± 2 to 105 ± 2 cm) but not in HIT (104 ± 1 to 103 ± 2 cm) and CON (104 ± 1 to 103 ± 1 cm). Waist circumference declined (*P* < 0.05) in HIT (86 ± 2 to 83 ± 2 cm) and MOD (94 ± 3 to 89 ± 3 cm) but was stable in CON (84 ± 2 and 82 ± 2 cm). Total body mass was lowered (*P* < 0.05) over 15 wks in HIT (76.5 ± 1.9 to 75.9 ± 2.1 kg) and MOD (83.8 ± 4.3 to 82.4 ± 4.0 kg), but remained similar in CON (76.4 ± 2.6 and 77.3 ± 2.2 kg) (Figure 2).

### 3.4. Plasma Cholesterol and Triglycerides

Total plasma cholesterol was 5.6 ± 0.2, 6.0 ± 0.2, and 5.3 ± 0.2 mmol·L^−1^ before the training intervention in HIT, MOD, and CON, respectively, and was similar after the intervention period (Table 1). HDL and LDL cholesterol was 1.4 ± 0.1 and 3.7 ± 0.2, 1.4 ± 0.1 and 3.9 ± 0.2, and 1.4 ± 0.1 and 3.5 ± 0.2 mmol·L^−1^ before training in HIT, MOD, and CON, respectively, and was unchanged after the intervention period (Table 1). Plasma triglyceride was 1.1 ± 0.1, 1.4 ± 0.1, and 1.0 ± 0.1 mmol·L^−1^ in HIT, MOD, and CON before training, but was unchanged after the training intervention (1.0 ± 0.1, 1.3 ± 0.2, and 1.3 ± 0.2 mmol·L^−1^).

### 3.5. Performance Testing

Before the training period, HIT and MOD covered 306 ± 15 and 305 ± 16 m, respectively, during the 10 min maximal swim. After the training period, performance was increased (*P* < 0.05) in HIT and MOD by 13 ± 3% and 22 ± 3%, respectively, resulting in a total distance of 342 ± 14 m and 368 ± 15 m (Figure 4(a)). The improvement in MOD tended (*P* = 0.07) to be higher than in HIT. Mean accumulated swimming time in the 4 × 25 m repeated sprint test was 153 ± 9 and 141 ± 5 s in HIT and MOD, respectively, before the intervention. After training, accumulated swimming time was reduced (*P* < 0.05) in both groups by 23 ± 3 and 8 ± 3%, respectively, reaching 116 ± 7 and 129 ± 6 s. HIT reduced their accumulated swimming time more (*P* < 0.05) than MOD (Figure 4(b)).

**Figure 3:**
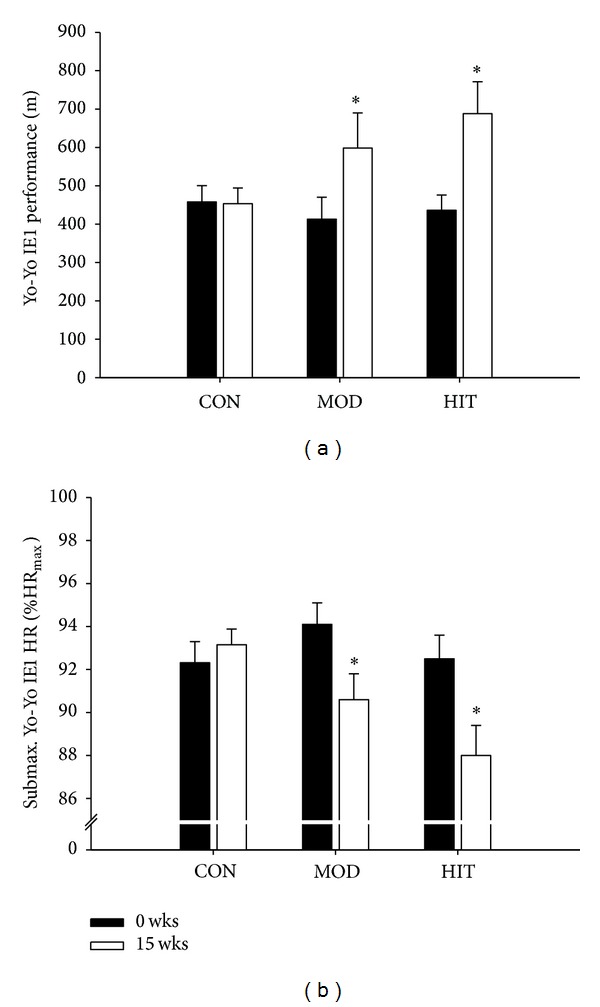
Yo-Yo Intermittent Endurance level 1 performance (a) as well as heart rate after controlled intermittent submaximal exercise (%HR_max⁡_, (b)) before and after 15 wks with thrice-weekly training sessions of high-intensity swimming (HIT) and moderate-intensity swimming (MOD) in comparison to an inactive control group (CON). Data are presented as means ± SEM. ∗ denotes significant difference between the training groups and CON.

**Figure 4:**
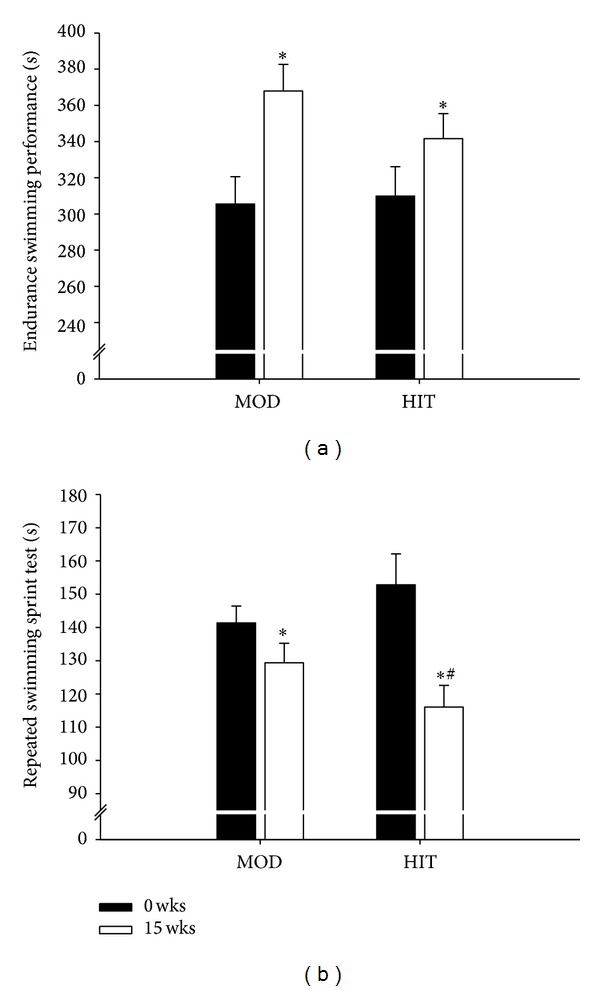
Endurance swimming performance (a) and repeated swimming sprint time (b) before and after 15 wks with thrice-weekly training sessions with high-intensity swimming (HIT) and moderate-intensity swimming (MOD) in comparison to an inactive control group (CON). Data are presented as means ± SEM. ∗ denotes significant difference between the training groups and CON. # denotes significant differences between HIT and MOD.

Yo-Yo IE1 performance before intervention was 413–458 m in the three groups (*P* > 0.05) and increased (*P* < 0.05) similarly by 58 ± 5 and 45 ± 4% after training in HIT and MOD, respectively, with no changes in CON (Figure 3(a)). HR after the initial five 2 × 20 m runs was 92.5 ± 1.1, 94.1 ± 1.0, and 92.3 ± 1.0% HR_max⁡_ before training in HIT, MOD, and CON, respectively, but was reduced (*P* < 0.05) similarly by 4.5 ± 0.5 and 3.5 ± 0.4% after intervention in HIT and MOD, respectively (88.0 ± 1.4 and 90.6 ± 1.2% HR_max⁡_, resp.) whilst HR remained similar in CON (93.2 ± 0.7% HR_max⁡_; Figure 3(b)). No difference was detected in shuttle-run performance or heart rate response between HIT and MOD.

## 4. Discussion

The present study is the first to examine if two different types of swim training can improve the cardiovascular health profile and land-based exercise capacity in sedentary premenopausal women with mild to moderate hypertension. The principal findings reveal that both short-term intermittent high-intensity and prolonged moderate-intensity swim training reduced systolic blood pressure and improved both water and land-based exercise capacities. Because the HI group only covered ~11–15% of the total mileage and spent one-third of time on training compared to the moderate-intensity training group, high-intensity intermittent training appears to be a time-efficient alternative to traditional recreational training regimes in untrained individuals.

The present findings support that swim training appears to have a high potential in the treatment of patients with arterial hypertension, which is supported by others applying aquatic training protocols [8–10, 28]. For example, similar decreases in blood pressure were reported by Tanaka et al. [28] and Nualnim and coworkers [10] demonstrated that 12 wks of regular swim training lowered SBP by 9 mmHg in adults older than 50 yrs with mild hypertension. In addition, the swim exercise training also produced a 21% increase in carotid artery compliance, as well as improvements in flow-mediated dilation and cardiovagal baroreflex sensitivity [10]. In addition, in a cross-sectional study, middle-aged swimmers had a lower carotid systolic blood pressure and carotid pulse pressure than sedentary controls. Moreover, carotid arterial compliance was higher and *β*-stiffness index was reported to be lower in the swimmers in comparison to the controls [29]. Thus, both intermittent high-intensity and continuous moderate-intensity swimming can be recommended for adults with mild hypertension.

In some review articles it is suggested that low-to-moderate-intensity exercise regimes are more efficient than protocols encompassing high-intensity exercise [30]. However, in the present study both training groups' SBP was lowered by 3-4%, which confirms findings in untrained men in their midthirties performing intense intermittent and continuous moderate-intensity running [6]. In the study by Nybo et al. [6], SBP was lowered in both training groups, but only DBP declined in the continuous moderate-intensity group, while mean arterial pressure (MAP) declined after both types of training. In the present study DBP was unchanged in both swim training groups, and MAP tended (*P* = 0.06) to decline only in the high-intensity training group with 76% of the participants demonstrating a reduction. Thus, the discrepancy between findings by Nybo et al. [6] and the present study may relate to the differences in training mode, since exercise in a supine position may provide a different training stimulus to cardiovascular parameters compared to upright exercise modes such as running due to the differences in ventricular volumes [31]. Moreover, gender differences have been shown to be present within a range of physiological adaptations to exercise training [4, 17]. Nybo et al. [6] used male participants and women seem to display smaller reductions in blood pressure after exercise training interventions in comparison to their male counterparts [4, 5, 19]. In contrast Ishikawa et al. [32] demonstrated that the gender did not influence the efficacy of physical activity for lowering elevated blood pressure.

In a recent study by Rocha et al. [33] isogenic rats were exposed to swim training at low, moderate, and high intensities and large morphological alterations in the cardiac myocytes occurred after high-intensity training in comparison to low and moderate intensities. These findings are in line with several recent review papers supporting that high-intensity training markedly reduced arterial blood pressure [18, 19, 34]. In addition, supportive of this notion are several studies on cardiovascular effects of recreational football training from our laboratory on sedentary men [5, 19, 23] and women [35, 36]. Thus, the rapid acceleration of heart rate and stroke volume during square-wave transitions from low- to high-intensity exercise as performed in the high-intensity training group in the present study may be an important stimulus to blood pressure reduction. This suggestion is backed up by the similar responses observed in the two training interventions despite large differences in training volume.

In the present study total fat mass was reduced and lean body mass increased in both training groups. In the aforementioned study by Nybo et al. [6] with untrained men high-intensity running did not change fat mass or fat oxidation during submaximal exercise. In contrast, the moderate-intensity running group displayed a reduction in fat mass as in the present study. Studies by Tjønna et al. [7, 37] and Schjerve et al. [38] compared “isocaloric” high- intensity and moderate-intensity training and found major advantages of the high-intensity training regimes. However, in these studies the overall energy turnover was matched in contrast to the present study. Therefore, it may be surprising that the reduction in fat mass was not different between the HIT and MOD interventions despite the large difference in total energy turnover. The caloric intake was not controlled in the present study, which may have affected the body fat adaptations. For example, it has recently been demonstrated that appetite regulating variables such as leptin are affected by high-intensity training [39]. Additionally, the findings in the present study are supported by others showing marked decreases in body fat content after high-intensity training [40–42].

No changes were observed in blood lipid profile in the present study, which is in contrast to findings by others demonstrating that prolonged moderate-intensity running reduces total/HDL-cholesterol ratio and elevates fat oxidation during exercise in contrast to brief intense interval training [6]. It is suggested that changes in blood lipid profile relates to changes in fat mass [43], and in the present study no statistical differences were evident between the reduction in body fat between the two interventions, which may partly explain the similar blood lipid responses. One explanation for the above-mentioned discrepancy might be that some of the participants in the present study had normal plasma cholesterol levels prior to the intervention. If the participants who had total cholesterol levels lower than 5.5 mmol·L^−1^ were excluded from the statistical analysis, there was a significant reduction in total cholesterol in the MOD training group and a tendency (*P* < 0.06) to a reduction in HIT (data not shown), indicating that women with high plasma cholesterol levels are more likely to respond to exercise.

The improved performance after high-intensity as well as moderate-intensity swimming conducted in the present study may be related to an improved physiological capacity, improved swimming technique, or both. The rather large ~50% improvement in land-based shuttle-run performance observed in the two swim-training groups, but not in the control group, strongly indicates that physiological adaptations are a major contributor for the augmented exercise capacity observed during both shuttle runs and swim tests. This is further supported by the ~4% heart rate reduction observed after the initial five 2 × 20 m runs indicating improved aerobic capacity. Previously, the possible transfer effect between swim training and land-based activity has been neglected. This is largely because increases in maximal oxygen uptake after swim training appear to be specific for that exercise modality as observed in monozygotic twins [44] and an observation of unchanged running VO_2_max, despite increased swimming VO_2_max in elite swimmers after 9 months of intense training [9, 45]. However, shuttle-run performance is not strongly correlated to VO_2_max [46, 47] and the current observation suggests that possible beneficial health adaptations obtained after swim training also translate into improved land-based exercise capacity. However, it also appears likely that a technical improvement may have occurred during the swim training. It can be speculated that the much higher total distance covered by the MOD group caused greater technical improvements than in the HIT group and that this is the reason for the tendency to a larger performance gain in the 10 min swim test of the MOD group. The larger improvement observed for the HIT than MOD group in repeated sprint swimming ability could be related to more specific motor learning leading to larger improvements in sprint technique in this group but could also be due to metabolic and physiological adaptations specific to sprinting. For example, high-intensity training is known to recruit more fast type II muscle fibres [48, 49] which could have yielded greater muscle hypertrophy and more pronounced enzymatic adaptations in type II fibres for the high-intensity group compared to the moderate-intensity training group [21]. Physiological adaptations that would favor improvements in intense short-duration exercise performance such as in the interval sprint test may be part of the explanation for the greater effect on sprint performance in the high-intensity swimming group (23 versus 8%). However, these suggestions remain speculative and warrant further investigation.

In conclusion, high-intensity intermittent swimming is a time-efficient and effective training method and improves cardiovascular health and physical performance in sedentary, premenopausal women with mild hypertension. Adaptations are similar with high- and moderate-intensity training, despite less total time spent and distance covered in the high-intensity group.

## Figures and Tables

**Table 1:** Plasma total, HDL, and LDL cholesterol (mmol·L^−1^) before and after a 15 wks intervention period in HIT, MOD, and CON.

	Total cholesterol		HDL		LDL	
	Before	After	Before	After	Before	After
HIT	5.6 ± 0.2	5.5 ± 0.2	1.4 ± 0.1	1.4 ± 0.1	3.7 ± 0.2	3.6 ± 0.2
MOD	6.0 ± 0.2	5.8 ± 0.3	1.4 ± 0.1	1.5 ± 0.1	3.9 ± 0.2	3.8 ± 0.3
CON	5.3 ± 0.2	5.4 ± 0.2	1.4 ± 0.1	1.3 ± 0.1	3.5 ± 0.2	3.4 ± 0.2

Data are expressed as means ± SEM.
